# 慢性粒-单核细胞白血病合并T淋巴母细胞淋巴瘤1例报告并文献复习

**DOI:** 10.3760/cma.j.cn121090-20250407-00163

**Published:** 2025-12

**Authors:** 玉泽 杨, 文艳 徐, 一桀 焦, 振兴 郭

**Affiliations:** 清华大学临床医学院，清华大学第一附属医院血液肿瘤科，北京 100016 Department of Hematology/Oncology, The First Hospital of Tsinghua University, School of Clinical Medicine, Tsinghua University, Beijing 100016, China

## Abstract

慢性粒-单核细胞白血病（CMML）与T淋巴母细胞淋巴瘤（T-LBL）均为血液系统恶性肿瘤，二者共存罕见。本文报道1例65岁女性患者，因“枕颈部肿块伴发热、盗汗1个月”就诊，经骨髓穿刺、淋巴结活检、流式细胞术及分子学检测，确诊为CMML-1合并T-LBL。患者在接受VDCP（长春地辛+柔红霉素+环磷酰胺+地塞米松）方案化疗后T-LBL达完全缓解，但后续复发并进展为T淋巴母细胞白血病（T-ALL）。在挽救治疗失败后，采用维奈克拉联合CHG方案诱导治疗，T-ALL再次达完全缓解，而CMML始终处于惰性病程。本文结合文献复习，探讨CMML与T-LBL共存的临床病理特征、分子机制及治疗策略，强调多学科协作与个体化治疗在罕见血液肿瘤中的重要性。

## Abstract (en)

The coexistence of chronic myelomonocytic leukemia（CMML）and T-lymphoblastic lymphoma (T-LBL) is extremely rare. We report a 65-year-old female patient who presented with occipitocervical masses, fever, and night sweats for 1 month. Comprehensive examinations, including bone marrow aspiration, lymph node biopsy, flow cytometry, and molecular profiling, confirmed the diagnosis of CMML-1 combined with T-LBL. The patient achieved complete remission of T-LBL after VDCP chemotherapy（vindesine, daunorubicin, cyclophosphamide, and dexamethasone）, but later relapsed and progressed to T-lymphoblastic leukemia（T-ALL）. After unsuccessful salvage therapies, she was treated with venetoclax combined with the CHG regimen, which induced a second complete remission of T-ALL. Notably, the CMML remained indolent throughout the clinical course. This article, supplemented with a literature review, emphasizes the clinicopathological features, molecular mechanisms, and therapeutic strategies for this rare coexistence, thereby highlighting the importance of multidisciplinary collaboration and individualized management in rare hematologic malignancies.

慢性粒-单核细胞白血病（CMML）是一种兼具骨髓增生异常综合征（MDS）和骨髓增殖性肿瘤（MPN）特征的克隆性造血干细胞疾病，以持续性单核细胞增多及多系发育异常为特征[1]。T淋巴母细胞淋巴瘤（T-LBL）则是起源于前体T淋巴细胞的侵袭性恶性肿瘤，常见于青少年，老年患者罕见[2]。CMML与T-LBL合并发生少有报道，其临床病理特征、分子机制及治疗策略尚不明确。现报告我院收治的1例CMML合并T-LBL患者，并对相关文献进行复习，以提高对此疾病诊疗的认识。

## 病例资料

患者，女，65岁，主因“枕颈部肿块伴发热、盗汗1个月”于2022年7月27日入住我院血液肿瘤科。患者2022年6月发现左枕部肿块，花生粒大小，伴轻度疼痛，低热、夜间盗汗，体重下降2 kg，未诊治。后因枕部肿块逐渐增多至颈部，转颈轻度受限，于2022年7月14日就诊于我院皮肤科门诊，血常规：WBC 11.15×10^9^/L，HGB 123 g/L，PLT 194×10^9^/L，单核细胞绝对值2.46×10^9^/L，单核细胞百分比21.3％。给予盐酸克林霉素棕榈酸酯分散片300 mg每日4次，疼痛减轻，但肿块数量进一步增多，腹股沟、腋下可见数枚肿物。患者既往有10年高血压病史，血压最高为150/80 mmHg（1 mmHg＝0.133 kPa），未规律服用降压药物。1年冠心病史，未用药。无染发史，否认放射性接触史，家族史无特殊。

查体：体温37.2 °C，脉率84次/min，呼吸20次/min，血压130/55 mmHg，身高152 cm，体重45 kg。枕后、颈部及双侧腹股沟、腋下多枚淋巴结肿大，较大淋巴结位于右侧腋下，约3.0 cm×2.0 cm，质硬，边缘清楚，活动度差，无触痛，无融合、粘连。胸骨无压痛，心肺未见异常。腹软，肝脾肋下未及，双下肢无水肿。入住血液肿瘤科后进一步完善相关检查，血常规：WBC 10.31×10^9^/L，HGB 113 g/L，PLT 112×10^9^/L，单核细胞绝对值2.28×10^9^/L，单核细胞百分比22.1％。颈、胸、腹部CT示双侧颈部、腮腺、腋下、腹股沟区及纵隔、右侧心膈角、腹腔内、腹膜后多发肿大淋巴结。骨髓细胞形态学：增生明显活跃，粒∶红为7.73∶1。粒系占58％，其中原始髓系细胞占有核细胞6％；单核细胞占12％，其中幼稚单核细胞占2％。粒细胞可见Pelger-Hüet畸形和核分叶过多，可见类巨变，嗜酸性、嗜碱性粒细胞比例增高，分别为5％和9％。骨髓细胞化学染色：中性粒细胞碱性磷酸酶（NAP）阳性率为61％，积分为76；髓过氧化物酶（MPO）染色阳性率为30％，呈弱至中等阳性。外周血涂片：原始细胞占2％，单核细胞占21％，个别细胞形态偏幼稚，嗜碱性粒细胞比例为10％。诊断意见：不除外CMML。

骨髓流式细胞术免疫表型：CD34阳性细胞占全部细胞的1.30％，表达CD33、CD38和CD7，部分表达CD117、CD13、CD123、HLA-DR和CD56，不表达CD11b、CD16、CD15、CD10、CD14、CD64、CD300e、CD36和CD19。胞内染色MPO、CD79a和CD3阴性。单核细胞及嗜碱性粒细胞比例增高，不排除MDS/MPN。骨髓染色体核型分析：46, XX, add（7）（p15）[5]/46, XX[15]。骨髓分子生物学检测：多重PCR筛查白血病融合基因为阴性；二代测序检测到NRAS p.G12D变异［变异等位基因频率（VAF）39.22％］、DNMT3A p.R882H变异（VAF 38.77％）及ETV6 c.1153-5_1153-1delAACAG变异（VAF 26.39％）。骨髓活检病理：粒、单核细胞增生（图1），符合CMML；免疫组化结果显示单核细胞表达LYS（弥漫阳性）及CD14（部分阳性），红系细胞表达CD71，髓系及淋巴系前体细胞散在表达CD34、CD117，淋巴细胞及浆细胞散在表达CD3、CD20、PAX5、CD138；网状纤维染色、铁染色、糖原染色阳性。

**图1:**
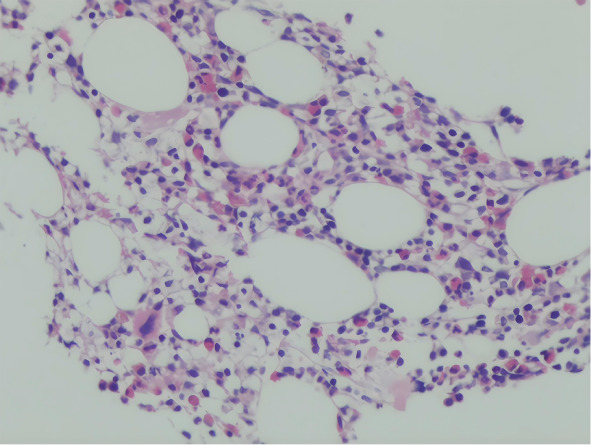
慢性粒-单核细胞白血病合并T淋巴母细胞淋巴瘤患者骨髓活检病理（HE染色，×200）

右侧腋窝淋巴结活检病理：淋巴结中见异常幼稚肿瘤细胞弥漫增生浸润（图2）；免疫组化结果显示表达原始淋巴细胞标志物CD34与TdT，以及T细胞标志物CD7、CD5与CD4；部分表达髓系标志物CD33、MPO、CD14、LYS及PG-M1；不表达B细胞标志物CD20与PAX5。Ki-67阳性率约60％，上述表型提示伴有髓系和T系分化特点，符合淋巴造血系统幼稚细胞肿瘤诊断特征。淋巴结流式细胞术免疫表型：异常幼稚T淋巴细胞占全部细胞的31.15％，表达CD5、CD7、CD99、TdT、CD56、CD33和CD38，部分表达CD13、CD123、HLA-DR，不表达CD2、CD3、CD4、CD8、CD1a、CD16、CD117、CD11b、CD15、CD64、CD14、CD36、CD300e、CD10、CD20和CD19，考虑为T-LBL伴髓系表达。综上，患者最终被确诊为CMML（世界卫生组织分型为CMML-1型），其CMML临床/分子预后积分系统（CPSS-mol）评分为2分（中危2组），梅奥分子模型（MMM）评分为4分（中危2组）；合并T-LBL，为Ann Arbor Ⅲ期，国际预后指数（IPI）评分为2分（低中危组）。

**图2:**
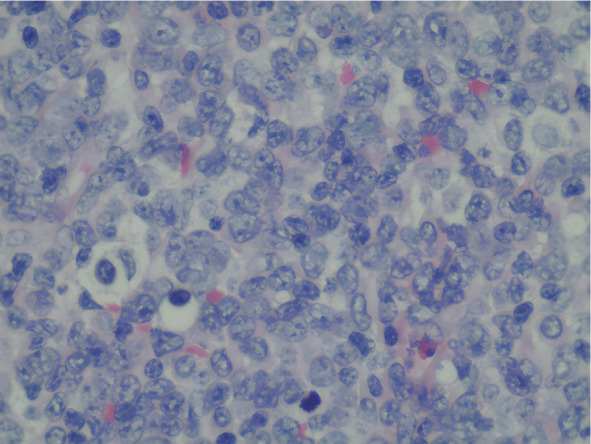
慢性粒-单核细胞白血病合并T淋巴母细胞淋巴瘤患者右侧腋窝淋巴结活检病理（HE染色，×400）

鉴于患者CMML临床进程相对缓慢，予以观察处理。而侵袭性T-LBL进展迅速，亟需紧急干预。因此，参照T-LBL治疗原则，患者于2022年8月17日起接受VDCP（长春地辛+柔红霉素+环磷酰胺+地塞米松）方案诱导化疗，腰椎穿刺鞘内注射防治中枢神经系统白血病。患者经2个周期治疗后疗效评价为部分缓解，骨髓流式细胞术微小残留病（MRD）阴性；颈、胸、腹部CT显示靶淋巴结径线乘积总和缩小66.3％。后接受中剂量阿糖胞苷、大剂量甲氨蝶呤以及VDCP方案巩固强化治疗5个疗程，PET-CT示全身未见淋巴瘤相关性代谢增高灶（Deauville评分2分），符合完全代谢缓解。疗效评价达完全缓解。2023年4月24日起行VMMP（长春地辛+巯嘌呤+甲氨蝶呤+泼尼松）方案维持治疗，多次复查均处于完全缓解状态。巩固强化及维持治疗期间（2022年10月至2024年2月），CMML相关指标持续稳定，仍呈惰性临床病程。

2024年3月19日评估T-LBL疾病进展，骨髓流式细胞术检测可见2.15％（占全部细胞）异常幼稚T淋巴细胞伴髓系表达，骨髓活检示T-LBL骨髓侵犯。淋巴瘤基因检测：NRAS p.G12D变异（VAF 39.17％），DNMT3A p.R882H变异（VAF 43.71％）。2024年4月11日起先后应用VDCP方案、中剂量阿糖胞苷挽救化疗，疗效均不佳，于2024年6月17日进一步进展为T淋巴母细胞白血病（T-ALL），骨髓流式细胞术免疫表型可见39.91％（占全部细胞）异常幼稚T淋巴细胞伴髓系表达（幼稚细胞表达CD7、CD3、CD5和CD33，部分表达CD34和CD56，不表达CD4和CD8）。此时患者CMML仍保持相对惰性状态。2024年6月21日给予患者维奈克拉联合CHG方案诱导化疗，具体为维奈克拉100 mg第1天、200 mg第2天、400 mg第3至10天；阿糖胞苷12.5 mg每12小时1次，皮下注射，第1至10天；高三尖杉酯碱1 mg，第1至10天；重组人粒细胞刺激因子注射液0.3 mg，第0至9天；硼替佐米1.5 mg，第1天、第8天；地塞米松10 mg，第1、2、8、9天。2024年6月30日复查骨髓流式细胞术MRD提示异常幼稚T淋巴细胞比例仅为0.02％（占全部细胞），疗效评价达完全缓解。此时，血常规提示单核细胞百分比16％，骨髓涂片偶见幼稚淋巴细胞，未见髓系原始细胞。提示该方案在清除T-ALL克隆的同时，维持了CMML的疾病稳定。患者目前继续治疗随访中。

## 讨论及文献复习

CMML与T-LBL均为血液系统恶性肿瘤，但二者在发病机制、病理特征及治疗策略上存在显著差异。本例患者骨髓分子学检测发现NRAS p.G12D、DNMT3A p.R882H变异，同时伴有ETV6剪切位点变异。NRAS变异在CMML和T-LBL中均常见，相对突变频率分别为15％[1]和10.8％[3]，其可通过激活MAPK通路促进肿瘤增殖。Wang等[4]研究表明，NRAS变异可能以突变负荷依赖的方式，参与CMML和T-LBL/ALL发生。DNMT3A突变在CMML中的相对频率为5％，通过表观遗传调控（DNA甲基化）影响造血干细胞分化[1]。T-LBL的分子特征以T细胞受体基因重排及NOTCH通路异常为主[5]，本例未检测到相关突变，淋巴结免疫表型显示肿瘤细胞TdT、CD7、CD56表达阳性，并伴髓系标志物CD33、CD13共表达。虽然少数T-LBL本身也可异常表达CD13、CD33等髓系标志物[6]，但结合本例患者同时存在具备NRAS、DNMT3A特征性突变的CMML克隆，上述免疫表型提示肿瘤细胞可能具有双系分化潜能。此外，本例患者骨髓检出克隆性染色体异常add（7）（p15），但在初诊T-LBL的淋巴结病灶中未被检出，因此，需进一步行FISH检查明确细胞起源。但由于是回顾性研究，标本量不足，无法行FISH。值得注意的是，在疾病进展为T-ALL后，骨髓分子学检测仍稳定维持原始CMML克隆的特征性突变谱（NRAS p.G12D/DNMT3A p.R882H）。上述时空异质性及突变谱的稳定性为两种肿瘤的独立克隆共存提供了关键证据。尽管如此，CMML与T-LBL的克隆关系仍需通过单细胞测序进一步验证。

CMML的典型表现为外周血单核细胞增多及骨髓发育异常[1]，而T-LBL常以淋巴结肿大或纵隔肿块为首发症状[6]。在诊断时，需与反应性单核细胞增多症及其他MDS/MPN亚型鉴别[7]，而且需排除混合表型急性白血病。本例通过流式细胞术明确髓系与淋系克隆的时空分离，排除了上述推断。值得注意的是，CMML患者合并快速进展的淋巴结肿大，易被误诊为继发感染或髓外急变。本例通过多部位活检及相应检测排除此类推断，强调了多学科协作的重要性。

CMML合并T-LBL罕见，目前尚缺乏临床管理共识。CMML标准治疗以去甲基化药物（HMA）为主，总反应率为40％～50％，完全缓解率<20％[8]。约60％的患者HMA治疗失败且与不良预后有关[9]。allo-HSCT仍是唯一可能治愈CMML的治疗方法，但受限于患者年龄、合并症及供体匹配问题[10]。T-LBL对急性淋巴细胞白血病（ALL）化疗方案敏感，在成人中治愈率高达70％[11]。但复发率高，复发后患者长期生存率不足20％[6]。近年来，BCL2抑制剂通过诱导肿瘤细胞凋亡在血液肿瘤治疗中展现出广阔前景。Cai等[12]通过小鼠模型发现调节BCL蛋白表达会影响CMML的疾病进程。Montalban-Bravo等[13]回顾性研究显示，27例接受维奈克拉联合方案治疗的CMML患者总缓解率达67％，支持该方案作为allo-HSCT前的有效桥接方案。

McBride等[14]研究表明ALL细胞凋亡依赖于BCL家族蛋白。一项多中心Ⅰ期临床研究评估了维奈克拉联合纳维托克在复发/难治ALL患者中耐受性良好，总缓解率为59.6％[15]。本例初始采用VDCP方案治疗，巩固强化后达完全缓解，缓解持续时间为12.5个月，但之后复发并快速进展为T-ALL。值得注意的是，尽管并未针对CMML进行特殊治疗，但在整个病程中，CMML克隆始终维持相对惰性的状态，而T-LBL克隆的侵袭性则主导了疾病进展，allo-HSCT可能是潜在有效的治疗策略。本例患者因客观条件放弃移植，在多线挽救治疗失败后应用维奈克拉联合CHG方案获得完全缓解，可能与维奈克拉靶向BCL2，从而克服肿瘤细胞凋亡抵抗的作用相关，但其长期疗效仍需随访确认。

综上所述，我们首次报道1例罕见的CMML与T-LBL共存病例，并通过多参数检测明确诊断。未来需通过多中心合作积累更多此类罕见病例，以进一步提高临床认识水平。
